# Spatial variation and socio-economic determinants of Plasmodium falciparum infection in northeastern Tanzania

**DOI:** 10.1186/1475-2875-10-145

**Published:** 2011-05-25

**Authors:** Bruno P Mmbando, Mathias L Kamugisha, John P Lusingu, Filbert Francis, Deus S Ishengoma, Thor G Theander, Martha M Lemnge, Thomas H Scheike

**Affiliations:** 1 National Institute for Medical Research, Tanga Centre, Tanzania; 2 Centre for Medical Parasitology, Institute for Medical Microbiology and Immunology, University of Copenhagen, Denmark; 3 Department of Biostatistics, University of Copenhagen, Denmark

## Abstract

**Background:**

Malaria due to *Plasmodium falciparum *is the leading cause of morbidity and mortality in Tanzania. According to health statistics, malaria accounts for about 30% and 15% of hospital admissions and deaths, respectively. The risk of *P. falciparum *infection varies across the country. This study describes the spatial variation and socio-economic determinants of *P. falciparum *infection in northeastern Tanzania.

**Methods:**

The study was conducted in 14 villages located in highland, lowland and urban areas of Korogwe district. Four cross-sectional malaria surveys involving individuals aged 0-19 years were conducted during short (Nov-Dec) and long (May-Jun) rainy seasons from November 2005 to June 2007. Household socio-economic status (SES) data were collected between Jan-April 2006 and household's geographical positions were collected using hand-held geographical positioning system (GPS) unit. The effects of risk factors were determined using generalized estimating equation and spatial risk of *P. falciparum *infection was modelled using a kernel (non-parametric) method.

**Results:**

There was a significant spatial variation of *P. falciparum *infection, and urban areas were at lower risk. Adjusting for covariates, high risk of *P. falciparum *infection was identified in rural areas of lowland and highland. Bed net coverage levels were independently associated with reduced risk of *P. falciparum *by 19.1% (95%CI: 8.9-28.2, p < 0.001) and by 39.3% (95%CI: 28.9-48.2, p < 0.001) in households with low and high coverage, respectively, compared to those without bed nets. Households with moderate and lower SES had risk of infection higher than 60% compared to those with higher SES; while inhabitants of houses built of mud walls were at 15.5% (95%CI: 0.1 - 33.3, p < 0.048) higher risk compared to those living in houses built by bricks. Individuals in houses with thatched roof had an excess risk of 17.3% (95%CI: 4.1 - 32.2, p < 0.009) compared to those living in houses roofed with iron sheet.

**Conclusions:**

There was high spatial variation of risk of *P. falciparum *infection and urban area was at the lowest risk. High bed net coverage, better SES and good housing were among the important risk factors associated with low risk of *P. falciparum *infection.

## Background

Malaria due to *Plasmodium falciparum *is a major public health problem in Sub-Saharan Africa and accounts for about 90% of malaria disease burden in the world. It has been estimated to cost approximately $12 billion per year due to the devastating effects of the disease and it affects the social and economic development of the region [1]. In Tanzania, malaria is the leading cause of morbidity and mortality and it accounts for about 30% and 15% of hospital admissions and deaths, respectively [2].

Malaria transmission intensity varies with season and decreases with altitude due to its dependence on temperature which affects the development of vectors and parasites [3]. High level of urbanization is also associated with less malaria and this is attributable to high pollution levels which affects mosquito larvae development, good housing [4], better access to health facilities and high bed nets coverage [5,6]. Other factors which affects transmission of malaria include rainfall, topography, land use and socio-economic status (SES) [7]. Changes in natural environment has also been associated with changing malaria transmission [7].

Application of geographical information system (GIS) and spatial statistical methods are regarded as important tools in epidemiology to identify areas with increased risk of diseases and determine spatial association between disease and risk factors [8-11]. In studies of distribution of malaria for example, it is not possible to measure and display all the variables that are associated with malaria transmission in a given geographical area. The use of maps and spatial statistical methods makes it easy to identify and display the unmeasured effect as a spatial effect and show areas with unusual high rates of the disease. Thus, disease-specific maps play an important role in disease control activities including monitoring the changes of the disease epidemiology, guiding resource allocation as well as identifying areas for further investigation [11,12].

The main objective of the study was to determine spatial variation and socio-economic determinants of *P. falciparum *infection in Korogwe district northeast Tanzania. Data from cross-sectional studies conducted between November 2005 and June 2007 in 14 villages of Korogwe district were used.

## Materials and methods

### Study area

**Figure 1:**
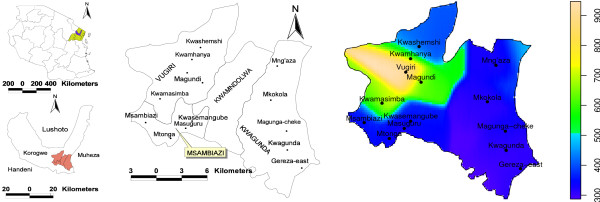
**Map showing wards (in capitals) and study villages (in lowercase) in Korogwe District (centre map)**. Top left, is the map of Tanzania showing location of Korogwe district within the Tanga region, while the bottom left, is the map of Korogwe district showing the location of the study villages. Coloured map shows elevation of the study area (metres above sea level) as calibrated in the legend

The study was conducted in 14 villages in Kwagunda, Msambiazi and Vugiri wards of Korogwe district in Tanzania (Figure 1) and individuals aged 0-19 years were involved. Selection of this age group was based on their variation in malaria immunity status [13]. The villages were chosen to represent areas with different malaria transmission intensities in preparation for sites for malaria vaccine trials. In this area, the major determinants of malaria transmission are altitude [3,14] and level of urbanization.

Since January 2006, this area has been under Demographic surveillance system (DSS), which is updated after every four months. A baseline census survey was conducted in November 2005 where households and demographic information for all members were registered. About 15% of the population in the study area were children aged below five years while 37% were individuals aged 5-19 years. Three of the study villages are located in Korogwe township (Mtonga, Masuguru and Kwasemangube) and the rest are in rural areas. The altitude of the area ranges from 300 to 1000mASL, (Figure 1) where four villages (Kwamasimba, Magundi, Kwamhanya and Vugiri) are located in the highlands (>500mASL). Six villages (Mkokola, Mng'aza, Kwashemshi, Kwamasimba, Magundi and Kwamhanya) have passive case detection (PCD) of febrile episodes where community health workers treats persons presenting with malaria symptoms by using first line anti-malaria drugs. These villages were selected in order to collect longitudinal data of febrile cases. Among the selection criteria was different altitude which is a proxy for transmission intensity in this area [15].

### Data collection

Four cross sectional malaria surveys were conducted in Nov-Dec 2005, May-Jun 2006, Nov-Dec 2006 and May-Jun 2007. In the study area, the period between May and June is characterized with high malaria transmission while Nov-Dec is a short transmission season. These periods correspond to the long (Mar-Jun) and short (Oct-Dec) rainy season. However, in the recent past there has been changes in the rainfall pattern.

About 40% of the children below five years of age and 15% of those aged 5-19 years were randomly selected from census database and adult individuals and parents/gurdians (for children) were sensitized to participate in the surveys. At each survey, demographic information of study participants were validated before physical examination and blood sampling. In brief, thick and thin smears were prepared from venous or finger prick blood, stained using Giemsa stain and read under a high power light microscope. For positive blood smears, parasites were counted against 200 white blood cells (WBCs) and a smear was declared negative after examining 100 high power fields. Household social economic data which included household properties, occupation of head of household and material used in construction of the house were collected during the first round of DSS (February-April 2006) using a questionnaire. Number of bed nets available and used in each household were collected during the first round of DSS, while information on whether an individual was using a bed net was sought during malaria cross-sectional surveys.

Mapping of households was done after the baseline census survey. Households positions (x, y coordinates) were captured using a hand-held global positioning system (GPS) receiver and were downloaded into a computer and transferred to ArcView 3.2 software. Distance between points (households) were converted from degrees into meters by multiplying longitude and latitude by 111,000 assuming a radial distance of the equator [16]. A map of the study area was prepared by overlaying the village coordinates in the map of the wards in ArcView. GIS data were then linked to malariometric and socio-economic data and transferred to the R statistical software http://cran.r-project.org for detailed analysis.

Ethical clearance was granted by the Medical Research Coordinating Committee of the National Institute for Medical Research, Tanzania. Informed consent was obtained during the survey, both orally and in writing from adult individuals while for children it was obtained from their parents or guardians.

### Statistical analysis

#### Scoring of socio-economic status

Principal component analysis was used to estimate the scores for the socio-economic status of the households. Some of variable which were considered in the principal component analysis were: source of power for lighting and cooking, ownership of radio, bicycle and mobile phone, ratio of number of sleeping bedrooms to number of household dwellers, occupation of head of household as well as number of animals and size of cultivated land owned by the family, see Table 1. Number of bed nets per household size and type of wall and roof of house were included as risk factors in modeling risk of *P. falciparum *infection. These variables were selected as a proxy for socio-economic status because is difficult to obtain household income information [17]. The SES scores were categorized into three groups (low, medium and high SES) at the ratio of 40:40:20 for easy interpretation of results [18,19].

**Table 1:** Variables used in assessing the socio-economic status (SES), their coding and estimated scores

Variable	Variable type and code	SES score
Source of power for lighting	Binary: (0 = Local lamp[koroboi], 1 = kerosene lump/electricity)	0.447
Source of power for cooking	Binary: (0 = Firewood, 1 = charcoal/kerosene/electricity)	0.402
Possess Mobile phone	Binary: (0 = No, 1 = yes)	0.390
Material used in making toilet walls	Binary: (0 = Thatch/mud walls, 1 = Bricks)	0.365
Ownership of bicycle	Binary: (0 = No, 1 = yes)	0.258
Occupation-formal employment	Binary: (0 = No, 1 = yes)	0.252
Source of water for domestic use	Ordinal: (1 = River, 2 = Well, 3 = Tap)	0.223
Availability of radio	Binary: (0 = No, 1 = yes)	0.210
Occupation - Small business	Binary: (0 = No, 1 = yes)	0.147
Number of sleeping rooms/Household size	Continuous [0-1][[Bibr B1]]	0.131
Ownership of house	Binary: (0 = No, 1 = yes)	0.127
Occupation - Petty business	Binary: (0 = No, 1 = yes)	0.092
Other occupation (such as masonry etc)	Binary: (0 = No, 1 = yes)	0.061
Number of cattle	Ordinal: (0 = none, 1 = 1-4, 2 = More than 4)	0.053
Number of goats/sheep	Ordinal: (0 = none, 1 = 1-10, 2 = More than 10)	-0.021
Number of chicken	Ordinal: (0 = None, 1 = 1-20, 2 = More than 20)	-0.025
Occupation - Peasant farmer	Binary: (0 = No, 1 = yes)	-0.226

### Modelling spatial variation of P. falciparum infection

In modeling spatial variation of *P. falciparum *infection, individuals who were found with *P. falciparum *parasite during the survey were considered as cases and those who were negative as multiple controls. Records of individuals were modeled as correlated observations since more than 50% of individuals had two or more observations in the four surveys. Let *π_ij _*be a probability of observing *Y_ij _*= 1 (*P. falciparum *case) in a person *i *= 1 ..., *n *seen in round *j *= 1 ..., *J *and living at location *x_i _*∈ *R*^2^; and let *Z_ij _*be a vector of length *p *of associated explanatory variables. Using marginal model, this was modeled as a binomial random variable:(1)

where *β *is a vector of *p *unknown regression parameters and *S*(*x_i_*): *x_i _*∈ *R*^2 ^is a smooth function for residence locations; which estimates the spatial effect across the study area. Non-parametric method was used in modeling the spatial effect as described by Kelsall and Diggle [20], modified to take into account the repeated observations within individuals. Generalized estimating equation (GEE) [21] was used in modeling the risk of malaria. The GEE is a solution to the score equation:(2)

where, Var(*Y_ij_*) = Var(*Y_ij_*, *β*, *α*), and *α *is the correlation between *Y_ij _*and *Y_ik_*. Estimation of *α *can be done by adding a second set of estimation equations *S_α_*(*β*, *α*) = 0 and solve the two equations simultaneously as detailed in Diggle *et al *[22]. Estimation of the odds of malaria was done in two parts: first by estimating the spatial component and then inserting the estimated spatial component into a model with other covariates and maximize iteratively. This was done by a back fitting procedure, where the spatial effect was estimated using gaussian kernel method (non-parametric). This was then inserted into GEE model using independent weights to obtain estimates of the parameters. The process of estimating parameters and spatial effect was done as follows:

Initialize *S*(*x_i_*) = 0, and estimate *β *using GEE

Step 1: set the link function 

• Construct an adjusted dependent variable  and weight 

Step 2: Fit an additive model  using a kernel regression with weights *w_ij _*as follows:

• set 

• perform a weighted kernel regression using weights *w_ij _*and estimate the spatial effect as: , where , is a spatial 2-dimensional kernel function and *h *is a bandwidth parameter.

• regress  on *z_ij _*using weighted GEE to obtain new values of .

Repeated step 1 and 2 until the estimates converge.

Bandwidth parameter *h *was estimated using cross validation technique which aims to find the values of *h *which minimizes:

where  is the estimate of *S^-i^*(*x_i_*) constructed with bandwidth *h *using all except the pair (*x_i_*, *μ_ij_*) [20].

To predict the risk of malaria in un-sampled locations, a set of new locations were generated at regular interval (nine locations at each of one by one squared kilometres), and then the spatial effect was estimated at each new location using the kernel method as described above. Spatial risk maps corrected for edge effect were generated using spatstat [23], effect of covariates were fitted by geepack and yags package was used to estimate the pseudo-AIC of different models. These packages are contained in R statistical software. Model with the lowest pseudo-AIC was selected as the best model. A gaussian kernel and a bandwidth parameter of 1,000 m estimated as detailed above, were used in the entire analysis. To assess the spatial uncertainty, parametric bootstrap sampling was used whereby 200 datasets of similar size to the original dataset were generated by random sampling of individuals' records with replacement and then fitting the model as described above. This gave 200 maps and these were used to generate 2.5% and 97.5% percentile maps.

## Results

A total of 12,298 data records of individuals from 14 villages collected during the four cross-sectional surveys were analyzed. Table 1 shows characteristic variables which were used to derive the socio-economic status of the households in the study area. Type of energy (for lighting and cooking) and possession of mobile phones were among the predictors of higher SES, while households headed by a peasant farmer were associated with lower SES. Individuals living in urban areas had higher SES than those living in highlands and rural lowlands. The mean scores of SES were 1.108 (95%CI: 1.046 - 1.169) in urban, -0.337 (95%CI: -0.369 - -0.304) in rural lowlands and -0.975 (95%CI: -1.013 - -0.935) in highlands. The pooled *P. falciparum *prevalence was 35.9 (95%CI: 34.9 - 36.9) in rural lowlands, 10.5 (95%CI: 9.3 - 11.7) in urban and 11.2 (95%CI: 9.7 - 12.7) in highlands.

### Spatial distribution of risk of P. falciparum infection

**Figure 2:**
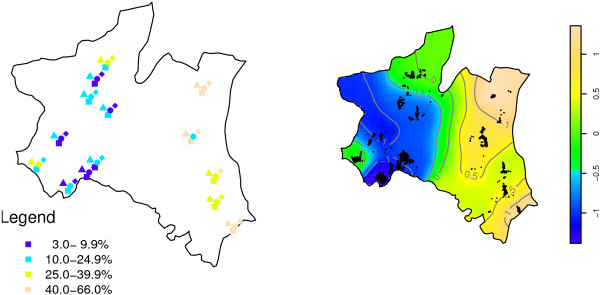
**Distribution of ranges of parasite prevalence (left) and smoothed un-adjusted risk of P. falciparum (right) during 2005-2007 cross-sectional surveys**. In the left, colours represents ranges of parasite prevalence as shown in the legend, while symbols shows survey 1-4 indicated by square, cycle, triangle and diamond, respectively. Smooth colors shows log odds ratio of *P. falciparum *infection, where deep blue and light brown show areas with lowest and highest risks, respectively. Point marks (right) shows residential location of individuals who were sampled during Nov 2005-May 2007 cross sectional surveys

Figure 2 shows distribution of *P. falciparum *parasite prevalence range during the four surveys and distribution of un-adjusted spatial log odds ratio for *P. falciparum *infection in the study area. The five villages on the right side of the map had higher malaria prevalence during the four surveys than the rest of the villages. A similar high risk surface in these villages is shown by un-adjusted smooth map, where north-eastern (Mng'aza) village and south-eastern (Gereza-east) village had log-odds ratio exceeding one when compared to the average map (i.e log odds ratio of zero). Villages in the urban and highlands had similar low risk of *P. falciparum *infection.

**Figure 3:**
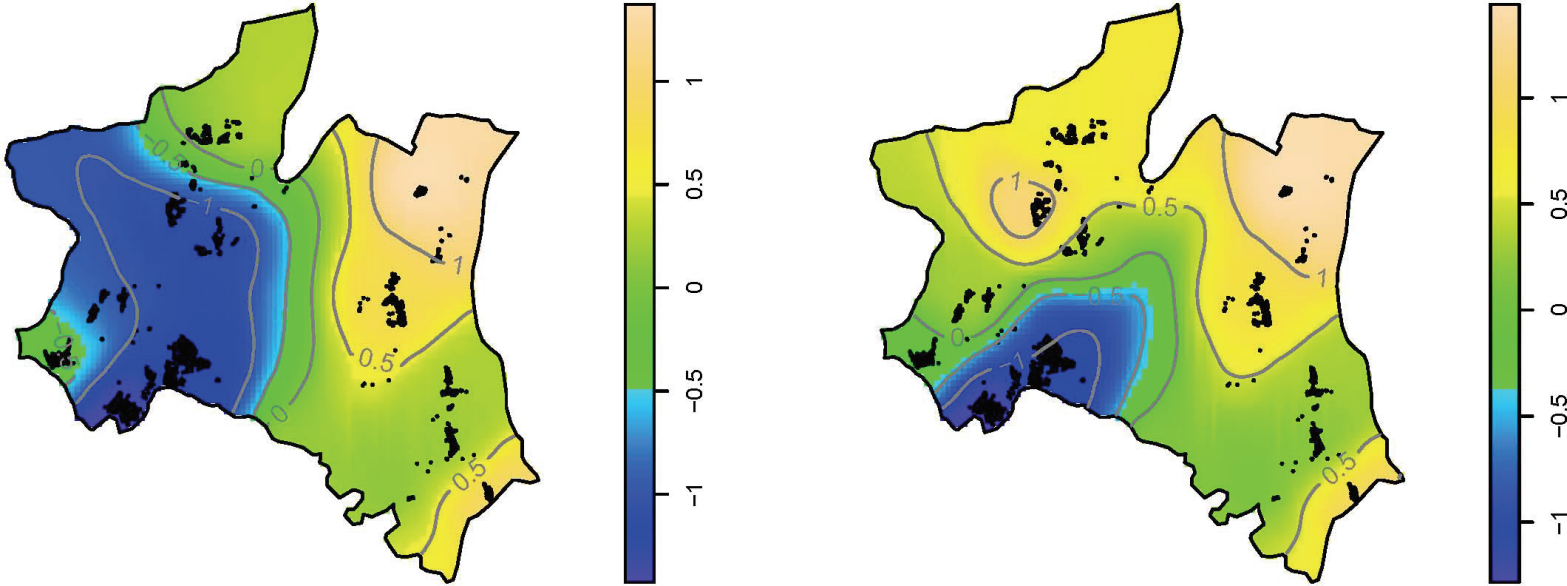
**Map showing the spatial variation in risk of P. falciparum infection in the study area**. Prediction based on model without altitude (left) and with altitude (right). Smooth colors shows log odds ratio of *P. falciparum *infection, where deep blue and light brown show areas with lowest and highest risks, respectively. Point marks shows residential location of individuals who were sampled during Nov 2005-May 2007 cross sectional surveys

When a model adjusted for risk factors (excluding altitude) was fitted, the maps showed a slightly different pattern of variation of risk of *P. falciparum *infection compared to un-adjusted map; for instance, the area with risk below log one increased to cover the three villages in the urban and two of the highland area, (Figure 3, left).

**Figure 4:**
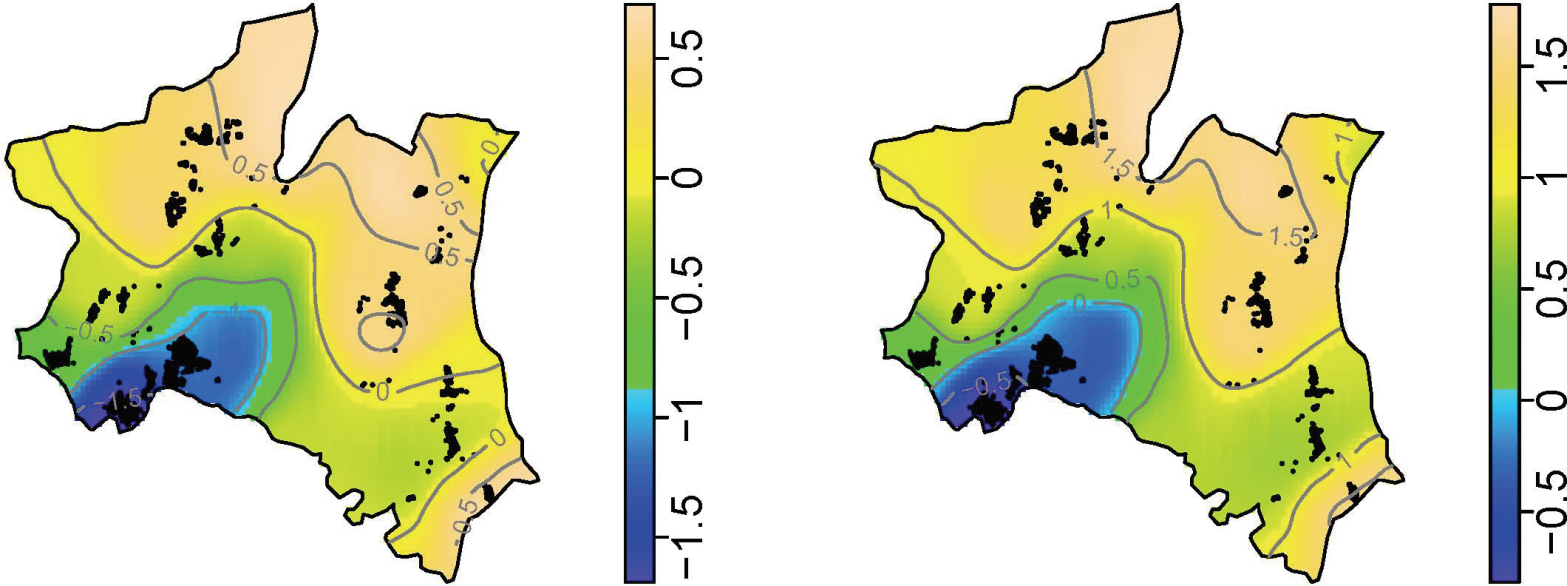
**Map showing 2.5% (left) and 97.5% (right) percentiles of risk of P. falciparum infection predicted from 200 bootstrap sampling adjusted for risk factors and altitude**. Smooth colors shows log odds ratio of *P. falciparum *infections, where deep blue and light brown show areas with lowest and highest risks, respectively.

Results from a model fitted with all risk factors including altitude, showed a shift in the risk of *P. falciparum *infection, whereby the urban area remained with low risk of infection, while the areas surrounding the urban were at a moderate risk. Villages which had highest risk remained the same, however, a village situated at the highest altitude (Vugiri) had significantly higher risk compared to the average map, (Figure 3, right). Maps of 2.5th and 97.5th percentiles from model fitted with all risk factors including altitude (Figure 4) showed that the estimates of spatial effects fitted in Figure 3(right) were consistent. The pseudo-AIC statistics showed that model with all covariates including altitude had lowest value (27,753.4) while one without covariate had the highest value (28,184.7), indicating that the full model was the best.

### Risk factors of P. falciparum infection

Table 2 shows results for variables that were associated with *P. falciparum *infection in the study area as fitted by a regression model outlined above. Individuals who were using bed nets had a 20.4% (95%CI: 11.7 - 28.4; p < 0.001) lower risk of infection than those not using nets. Compared to individuals living in households without bed nets and independent of current use of bed nets; individuals who were living in households where the ratio of bed nets to people was (0-0.4] (i.e a ratio greater than zero and ≤ 0.4), the risk of *P. falciparum *infection were reduced by 19.1% (95%CI: 8.9-28.2, p < 0.001) while in households with a ratio above 0.4, the risk was reduced by 39.3% (95%CI: 28.9-48.2, p < 0.001).

**Table 2:** Distribution of multiple regression parameter estimates for factors associated with risk of P. falciparum infection as estimated by equation (1), where the spatial component is presented in Figure (3, right)

Variable	OR	95%	CI	P-value
Age	1.41	1.345	1.477	<0.001
Age squared	0.983	0.980	0.985	<0.001
bed net use	0.796	0.716	0.883	<0.001
Bed net rate (Zero)	1			
Bed net rate (0-0.4]	0.809	0.718	0.911	<0.001
Bed net rate (0.4-1]	0.607	0.518	0.711	<0.001
SES -(High)	1			
Medium	1.605	1.336	1.927	<0.001
Low	1.611	1.359	1.910	<0.001
Type of roof (Thatch)	1.173	1.041	1.322	0.009
Type of wall (Mud walls)	1.155	1.001	1.333	0.048
Year -2005	1			
2006	0.853	0.747	0.974	0.019
2007	0.778	0.640	0.946	0.012
Short rains	1.124	0.995	1.269	0.059
PCD	0.760	0.680	0.851	<0.001
Altitude (by 100m)	0.664	0.640	0.689	<0.001

PCD = Precense of passive case detection, SES = Socio-economic status

Socio-economic status of household was also a significant predictor of malaria infection, where the risk was 60.5% higher (95%CI: 33.6-92.7, p < 0.001) in households with moderate and 61.1% higher (95%CI: 35.9 - 91.0, p < 0.001) in low SES compared to those with highest SES. Moreover, individuals living in houses built of mud walls had a 15.5% (95%CI:00.1 - 33.3, p < 0.048) higher risk compared to those living in houses built of bricks, while those who were living in houses with thatched roof were at a 17.3% (95%CI:4.1 - 32.2, p < 0.009) higher risk than those with houses roofed by iron sheets. Individuals living in villages with passive case detection were also at a reduced risk of *P. falciparum *infection by 24.0% (95%CI: 14.9 - 32.0, p < 0.001), Table 2.

## Discussion

The aim of this study was to determine the spatial variation and socio-economic determinants of *P. falciparum *infection in an area with different transmission intensities in northeastern Tanzania. Results from this study showed high spatial variability in the risk of *P. falciparum *infection. The highest risk was found in the lowland rural area, while the lowest risk was in the urban area [24,25]. The pattern of spatial variation in risk of *P. falciparum *infection were shown to vary in the maps adjusted for risk factors. A map adjusted for risk factors excluding altitude indicated a similar risk between the highlands and urban areas. This map had lower spatial heterogeneity in risk of *P. falciparum *infection than un-adjusted map. This shows that some of the variations which were seen in the un-adjusted map were due to risk factors included in the model.

The risk in highland area was shown to increase substantially when the effect of altitude which is a proxy for malaria transmission in this area was included in the model [15]. Inhabitants of one of the villages in the highland (Vugiri) were shown to have a risk almost similar to that of inhabitants of the lowland village (Mng'aza). Inhabitants of Vugiri village did not have passive case detection system (PCD); however, since the map was also adjusted for the effect of PCD, the risk would be expected to be similar to that of surrounding villages. This indicates that the transmission in this village could not be explained by variables included in the model.

The reason for high risk in Mng'aza and Mkokola villages which are located in the lowland area could be proximity to Lwengera River which flows on the eastern side of the two villages, while a stream which flows along the south-east border of Gereza-east village might be the reason for high infection in this village. These water bodies might be the source of the breeding sites for mosquitoes and hence increased transmission.

It is obvious that some of important risk factors such as use of mosquito repellents, management of surroundings, and distribution and distances to mosquitoes breeding sites were not accounted for in these models. Inclusion of these information could explain more of the observed spatial variation in the study area. Unfortunately, no recent entomological studies were done in the study area which could provide more information on mosquito breeding and transmission dynamics.

Socio-economic status of households was an important risk factor for malaria in the study area, where households of lower to moderate socio-economic status were at a more than 60% higher risk compared to households of highest socio-economic status. Similar findings have been reported elsewhere [6,26,27]. Poor housing was also among the risk factors for malaria in the study area, whereby individuals living in houses built of mud and thatched roofs were at a higher risk compared to individuals of similar socio-economic status but living in better houses. Similar findings were reported by a study in Sri Lanka where mosquito abundance was associated with the poor housing construction [28] and in South Africa where a case-control study showed a six fold risk of malaria in houses built of mud walls compared to bricks [27]. This indicates that better houses do not give easy entry and hiding places for mosquitoes.

High coverage of bed nets was an important finding in our study area; this was associated with reduction of infection regardless of whether an individual was using a bed net. For example, individual living in a household with five persons and two bed nets was at 20% lower risk of infection than those living in households without a net. This indicates the importance of high coverage of bed nets in reduction of malaria infection [29-31]. A study done in malaria endemic area of Tanzania [32], showed that the density of bed nets coverage in households was the only significant covariate associated with reduction in mortality, which is an important indicator in reduction of malaria burden.

## Conclusion

Results from this study showed high variability of the risk of malaria within the study area. Altitude, SES, high bed net coverage and urbanization are amongst the factors associated with the spatial variability in malaria. Improving access to means of protection against mosquitoes and living standards of the population might have a significant impact on the burden of malaria. The risk maps helped to identify areas with high risk of *P. falciparum *infection and this can be useful in malaria control in the area. Prediction maps outlined in this study can be used in priority settings; whereby the maps un-adjusted for altitude can be used to show areas with high risk where treatment should be diverted to, while maps adjusted for risk factors indicates areas where transmission is higher than would be expected and prevention might be the most effective. These map could also be used to identify areas where further investigation is needed to ascertain the cause of high risk of *P. falciparum *infection.

## Competing interests

The authors declare that they have no competing interests.

## List of abbreviations

ASL: Above sea level; DSS: Demographic surveillance system; GEE: Generalized estimating equation; GIS: Geographical information system; GPS: Global positioning system; OR: Odds ratio; PC: Principal component; PCA: Principal component analysis; PCD: Passive case detection; SES: Socio-economic status; WBC: White blood cells.

## Authors' contributions

BPM participated in designing the study, data collection and analysis, and manuscript writing. MLK and DSI participated in designing the study and data collection. JPA and MML participated in designing the study, supervision of field and laboratory work. FF-Management of data. TGT participated in designing the study and scientific suggestions. THS Participated in analysis design and commented in all stages of statistical analysis. All authors read and approved the final manuscript for publication.
